# SPOP inhibits BRAF-dependent tumorigenesis through promoting non-degradative ubiquitination of BRAF

**DOI:** 10.1186/s13578-022-00950-z

**Published:** 2022-12-30

**Authors:** Kai Feng, Qing Shi, Dongyue Jiao, Yingji Chen, Wanqi Yang, Ke Su, Yalan Wang, Yan Huang, Pingzhao Zhang, Yao Li, Chenji Wang

**Affiliations:** 1 Shanghai Stomatological Hospital & School of Stomatology, State Key Laboratory of Genetic Engineering, MOE Engineering Research Center of Gene Technology, Shanghai Engineering Research Center of Industrial Microorganisms, School of Life Sciences, Fudan University, Shanghai, 200438 China; 2 State Key Laboratory of Genetic Engineering, Human Phenome Institute, School of Life Sciences, Fudan University, Shanghai, 200438 China; 3 Obstetrics and Gynecology Hospital, NHC Key Laboratory of Reproduction Regulation, Shanghai Institute of Planned Parenthood Research, State Key Laboratory of Genetic Engineering, School of Life Sciences, Fudan University, Shanghai, 200438 China; 4 Department of Pathology, School of Basic Medical Sciences, Fudan University Shanghai Cancer Center, Fudan University, Shanghai, 200032 China

**Keywords:** SPOP, BRAF, Mutation, Ubiquitination, MAPK/ERK

## Abstract

**Background:**

The gene encoding the E3 ubiquitin ligase substrate-binding adapter Speckle-type BTB/POZ protein (*SPOP*) is frequently mutated in prostate cancer (PCa) and endometrial cancer (EC); however, the molecular mechanisms underlying the contribution of SPOP mutations to tumorigenesis remain poorly understood.

**Methods:**

BRAF harbors a potential SPOP-binding consensus motif (SBC) motif. Co-immunoprecipitation assays demonstrated that BRAF interacts with SPOP. A series of functional analyses in cell lines were performed to investigate the biological significance of MAPK/ERK activation caused by SPOP mutations.

**Results:**

Cytoplasmic SPOP binds to and induces non-degradative ubiquitination of BRAF, thereby reducing the interaction between BRAF and other core components of the MAPK/ERK pathway. SPOP ablation increased MAPK/ERK activation. EC- or PCa-associated SPOP mutants showed a reduced capacity to bind and ubiquitinate BRAF. Moreover, cancer-associated BRAF mutations disrupted the BRAF-SPOP interaction and allowed BRAF to evade SPOP-mediated ubiquitination, thereby upregulating MAPK/ERK signaling and enhancing the neoplastic phenotypes of cancer cells.

**Conclusions:**

Our findings provide new insights into the molecular link between SPOP mutation-driven tumorigenesis and aberrant BRAF-dependent activation of the MAPK/ERK pathway.

**Supplementary Information:**

The online version contains supplementary material available at 10.1186/s13578-022-00950-z.

## Introduction

The MAPK/ERK pathway, also known as the RAS-RAF-MEK-ERK pathway, transmits extracellular proliferative signals from the ligand-mediated activation of receptor tyrosine kinases to the cell nucleus [1, 2]. BRAF is a member of the rapidly accelerated fibrosarcoma (RAF) kinase family, which includes ARAF and CRAF (RAF1) [3]. RAFs transduce signals downstream of the RAS via the MEK-ERK cascade. Among the three RAF kinases, BRAF binds most avidly to RAS and is the most active in phosphorylating MEK1/2 [4]. Oncogenic BRAF mutations have been detected in approximately 6% of human cancers, with most occurring in hairy cell leukemia (> 97%), melanoma (40–50%), thyroid cancer (30–50%), colorectal cancer (10%), and non-small cell lung cancer (3–5%) [5]. In contrast, mutations in ARAF and CRAF are relatively rare in human cancers. Oncogenic BRAF mutations lead to the constitutive activation of the MAPK/ERK pathway, resulting in uncontrolled proliferation, survival, invasiveness, and drug resistance [1]. BRAF inhibitors such as vemurafenib, dabrafenib, and encorafenib are currently used to treat patients with BRAF-mutant cancers [6, 7].

Cullin-RING E3 ubiquitin ligases (CRLs) are a superfamily of modular, multi-subunit E3 ubiquitin ligases that mediate the ubiquitination and degradation of various cellular proteins involved in a plethora of physiological and pathological processes. Human cells express seven different Cullins (CUL1, 2, 3, 4A, 4B, 5, and 7), each of which nucleates a multi-subunit complex [8]. The Cullin3-RING E3 ubiquitin ligase complex subfamily employs a BTB domain-containing protein for substrate-binding. Speckle-type BTB/POZ protein (SPOP) is a substrate-binding adaptor of CRL3s [9]. Investigation of the pathophysiological functions of SPOP has attracted extensive attention in recent years because of its high mutation frequency in prostate cancer (PCa) (5–15%) and endometrial cancer (EC) (8–10%), two hormone-related cancers [10, 11]. Recurrent SPOP mutations have also been detected in other cancer types, although at a relatively low frequency. SPOP mutations occur as heterozygous missense mutations with dominant-negative, selective loss-of-function effects on the remaining wild-type allele [12]. SPOP selectively recruits substrates via its N-terminal MATH domain, whereas its BTB and BACK domains mediate oligomerization and interaction with CUL3 [9]. The vast majority of EC- or PCa-associated SPOP mutations occur in the substrate-binding MATH domain. To date, dozens of SPOP substrates (AR, ERα, SRC-3, BET, GLP, etc.) have been identified through different biochemical methods and functionally investigated in PCa or EC settings, and the list of SPOP substrates is still growing [13–17]. Research has shown that cancer-associated SPOP mutants generally display an impaired capacity to bind substrates, leading to reduced ubiquitination of substrates. Accumulating evidence from cancer cell lines and animal models has demonstrated that SPOP mutations promote the initiation and progression of EC and PCa, potentially owing to the dysregulation of SPOP-mediated ubiquitination of its substrates [15, 18]. However, the cellular pathways affected by SPOP mutations are not yet fully understood.

In this study, we identified BRAF as a *bona fide* substrate of the CRL3-SPOP E3 ubiquitin ligase complex. The CRL3-SPOP complex mediates non-degradative ubiquitination of BRAF, which leads to attenuated MAPK/ERK activation. Moreover, mutations in the SPOP-BRAF-binding interface disrupt the SPOP-BRAF regulatory pattern and promote aberrant MAPK/ERK activation and malignant transformation in cancer cells.

## Methods

### Cell culture

293 T, Ishikawa, KLE, SPEC-2, and HeLa cells were maintained in DMEM supplemented with 10% (v/v) fetal bovine serum (FBS). SPEC-2 cells were maintained in F12 medium supplemented with 10% (v/v) FBS. All cells were grown at 37 °C with 5% CO_2_.

### Plasmid constructions

Expression vectors for SPOP-WT and its mutants have been described previously [14]. BRAF cDNA was obtained from Genecopia and subcloned into the pCMV vector. BRAF mutants were generated using the KOD-Plus-Mutagenesis Kit (TOYOBO) following the manufacturer’s instructions.

### CRISPR-Cas9 mediated gene knock out stable cell generation

The pX459 plasmid was used to clone guide oligonucleotides targeting *BRAF* and *SPOP* genes. Ishikawa and SPEC-2 cells were plated and transfected overnight with pX459. 24 h after transfection, 1 μg/ml puromycin was used to screen cells for 3 days. Living cells were seeded in 96 well plate at a limited dilution to isolate the monoclonal cell lines. KO cell clones were screened by Western blot (WB) and validated by Sanger sequencing. The sequences of gene-specific sgRNAs are listed in Additional file 1: Table S1.

### Protein half-life assays

To measure protein half-life, cycloheximide (50 μg/ml) was added to the media of the parental and SPOP-KO Ishikawa cells. At the indicated time points, cell lysates were prepared and analyzed by WB using the indicated antibodies.

### Colony formation assays

Ishikawa cells (1 × 10^3^) were seeded in triplicate in 6-well plates containing each well. After incubation for 2 weeks, the cells were fixed with 4% paraformaldehyde for 15 min and stained with Giemsa dye (Solarbio) for 20 min. The cells were then washed with water by gentle dropping and air dried at room temperature. The number of colonies was quantified using a Nikon digital camera and the ImageJ software.

### In vivo ubiquitination assays

293 T cells were transfected with HA-Ubiquitin and other indicated constructs. 36 h after transfection, cells were lysed in 1% SDS buffer (Tris [pH 7.5], 0.5 mM EDTA, 1 mM DTT) and boiled for 10 min. For co-immunoprecipitation (Co-IP), cell lysates were diluted tenfold in Tris–HCl buffer and incubated with anti-FLAG M2 agarose beads (Sigma) for 4 h at 4 °C. The bound beads were then washed four times with BC100 buffer (20 mM Tris–Cl, pH 7.9, 100 mM NaCl, 0.2 mM EDTA, 20% glycerol) containing 0.2% Triton X-100. Proteins were eluted with 3X FLAG peptide for 2 h at 4 °C. The ubiquitinated form of BRAF was detected by WB with anti-HA antibody.

### In vitro ubiquitination assays

In vitro ubiquitination assays were performed according to a protocol reported previously [12]. Briefly, 2 µg APP-BP1/Uba3, 2 µg His-UBE2M, and 5 µg NEDD8 were incubated at 30 °C for 2 h in the presence of ATP. The thioester-loaded His-UBE2M–NEDD8 was further incubated with 3 µg His-DCNL2 and 6 µg CUL3–RBX1 at 4 °C for 2 h to obtain neddylated CUL3–RBX1. The neddylated CUL3–RBX1, 5 µg of GST-SPOP, 5 µg of ubiquitin, 500 ng of E1 enzyme, 750 ng of E2 enzyme (UbcH5a and UbcH5b), and 5 µg of GST-BRAF were incubated with 0.6 µl of 100 mM ATP, 1.5 µl of 20 µM ubiquitin aldehyde, 3 µL of 10 × ubiquitin reaction buffer (500 mM Tris–HCl (pH 7.5), 50 mM KCl, 50 mM NaF, 50 mM MgCl2, and 5 mM DTT), 3 µL of 10 × energy regeneration mix (200 mM creatine phosphate and 2 µg/µL creatine phosphokinase), and 3 µL of 10 × protease inhibitor cocktail at 30 °C for 2 h, followed by WB analysis. Recombinant proteins Information is presented in Additional file 1: Table S2.

### Cell proliferation assays

The proliferation rate of Ishikawa cells was determined using the Cell Counting Kit-8 (CCK-8) Kit (Beyotime) according to the manufacturer’s instructions. Briefly, the cells were seeded onto 96-well plates at a density of 1 × 10^3^ cells/well in triplicate. During the 2-to 6 day culture period, 10 μl of CCK-8 solution was added to the cell culture and incubated for 2 h. The absorbance of the samples was measured at 450 nm wavelength using a microplate absorbance reader.

### Migration and invasion assays

Cell migration and invasion were determined using Transwell (Costar) migration and invasion assays. Ishikawa cells were precultured in serum-free medium for 48 h. For the migration assay, 3 × 10^4^ cells were seeded in serum-free medium in the upper chamber, and the lower chamber was filled with DMEM containing 5% FBS. After 48 h, the non-migrating cells in the upper chambers were carefully removed with a cotton swab and migrated cells underside of the filter were stained and counted in nine different fields. Matrigel invasion assays were performed using Transwell inserts (Costar) coated with Matrigel/fibronectin (BD Biosciences).

### Sphere formation assays

Ishikawa cells (1 × 10^3^ cells/well) were mixed with Matrigel (BD Biosciences) and plated in 24-well ultra-low-attachment plates (Corning) in DMEM containing 10% FBS. Fresh medium was added every 3 days. Floating spheres that grew in 1–2 weeks were quantified using a Nikon digital photo camera and the ImageJ software.

### Immunofluorescence and confocal microscopy

For immunofluorescence, cells were plated on chamber slides and fixed with 4% paraformaldehyde at room temperature for 30 min. After washing with PBS, cells were permeabilized with 0.1% Triton X-100 in PBS for 15 min. The cells were then washed with PBS, blocked with 0.5% BSA in PBS for 1 h, and incubated with primary antibodies in PBS at 4 °C overnight. After washing with PBS, fluorescence-labeled secondary antibodies were applied and DAPI was counterstained for 1 h at room temperature. The cells were visualized and imaged using a confocal microscope (LSM710, Zeiss).

### Statistical analysis

Statistical analysis was performed using GraphPad Prism (GraphPad Software, Inc.), and all data are shown as mean values ± standard deviation (SD) for experiments performed with at least three replicates. The differences between the two groups were analyzed using one-way or two-way ANOVA test unless otherwise specified. * represents p < 0.05, ** represents p < 0.01, *** represents p < 0.001, **** represents p < 0.0001, n.s. represents non-significant.

## Results

### Identification of BRAF as a novel SPOP interactor

**Fig. 1:**
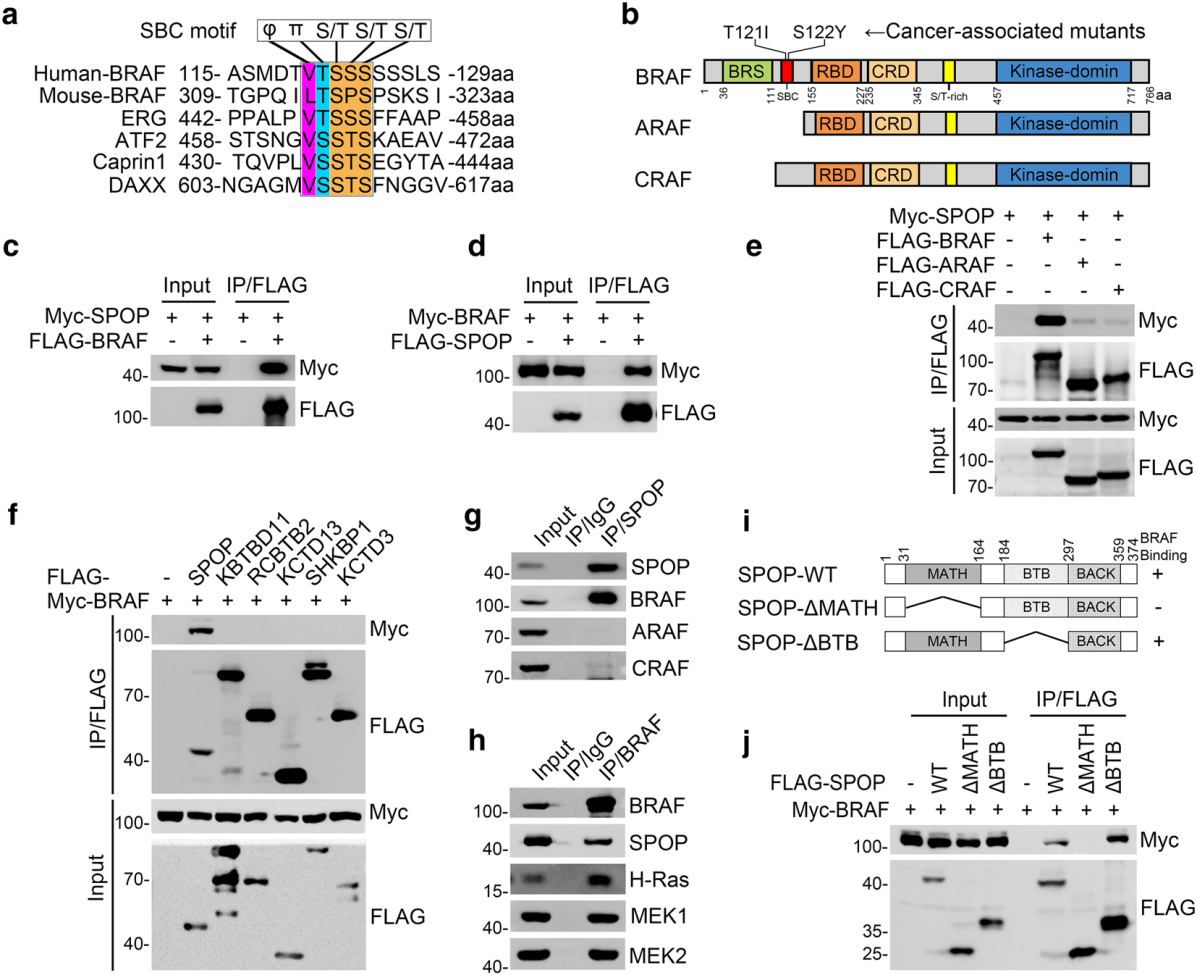
SPOP interacts with BRAF in cells. **a** Amino acid sequence alignment of the SBC motif in SPOP substrates. ERG, ATF2, Caprin1, and DAXX are reported SPOP substrates containing the well-characterized SBC motif (Φ-π-S–S/T-S/T; Φ: nonpolar residues, π: polar residues). **b** Diagram showing the RAF protein family, putative SBC motif, and two cancer-associated BRAF mutants. **c**–**e** Western blot of indicated proteins in whole-cell lysates (WCL) and co-IP samples of anti-FLAG antibody obtained from 293 T cells transfected with indicated plasmids. **f** Western blot of indicated proteins in WCL and co-IP samples of anti-FLAG antibody obtained from 293 T cells transfected with indicated plasmids. **g** Western blot of indicated proteins in WCL and co-IP samples of IgG or anti-SPOP antibody obtained from the cell extracts of Ishikawa cells. **h** Western blot of indicated proteins in WCL and co-IP samples of IgG or anti-BRAF antibody obtained from the cell extracts of Ishikawa cells. **i** Schematic representation of SPOP deletion mutants. The binding capacity of SPOP to BRAF is indicated with the symbol. **j** Western blot of indicated proteins in WCL and co-IP samples of anti-FLAG antibody obtained from 293 T cells transfected with indicated plasmids

Previously, we and others have identified multiple oncoproteins as degradative or non-degradative substrates of the CRL-SPOP complex using the yeast two-hybrid method or affinity purification coupled with mass spectrometry. Substrate-binding consensus (SBC) motifs (Φ-π-S/T-S/T-S/T, where Φ is a nonpolar residue and π is a polar residue) have been well-characterized in the majority, if not all, of these substrates [9]. To expand the SPOP interactome to a proteome-wide level, we performed an SBC motif search against the human proteome sequence using the webtool ScanProsite (https://prosite.expasy.org/scanprosite/). Among the hundreds of potential candidates identified by this search, we noticed that BRAF harbors a perfectly matched SBC motif (120-VTSSS-124 aa), which is very similar to those present in several known SPOP substrates, including ERG, ATF2, Caprin1, and DAXX (Fig. 1a). Notably, this motif was absent in BRAF paralogs of ARAF and CRAF (Fig. 1b). Given that BRAF is frequently hyperactivated in various human cancers, we explored whether BRAF is an authentic substrate of the CRL3-SPOP complex, and whether BRAF activity is dysregulated in SPOP-mutated cancers. Using reciprocal co-IP assays, we demonstrated that ectopically expressed BRAF interacted with SPOP (Fig. 1c, d). In contrast, ectopically expressed ARAF or CRAF did not interact with SPOP (Fig. 1e). Only SPOP, but none of the other CRL3-based BTB domain-containing adaptors examined, interacted with BRAF (Fig. 1f). Moreover, a specific endogenous interaction between SPOP and BRAF was observed in Ishikawa endometrial cancer cells (Fig. 1g, h). In accordance with a previous study showing that the MATH domain of SPOP is responsible for recruiting substrates [9], deletion of the MATH domain, but not the CUL3-binding BTB domain, completely abolished the interaction between SPOP and BRAF (Fig. 1i, j). Taken together, our data indicate that SPOP interacts with BRAF in cells.

### SPOP promotes non-degradative ubiquitination of BRAF

**Fig. 2:**
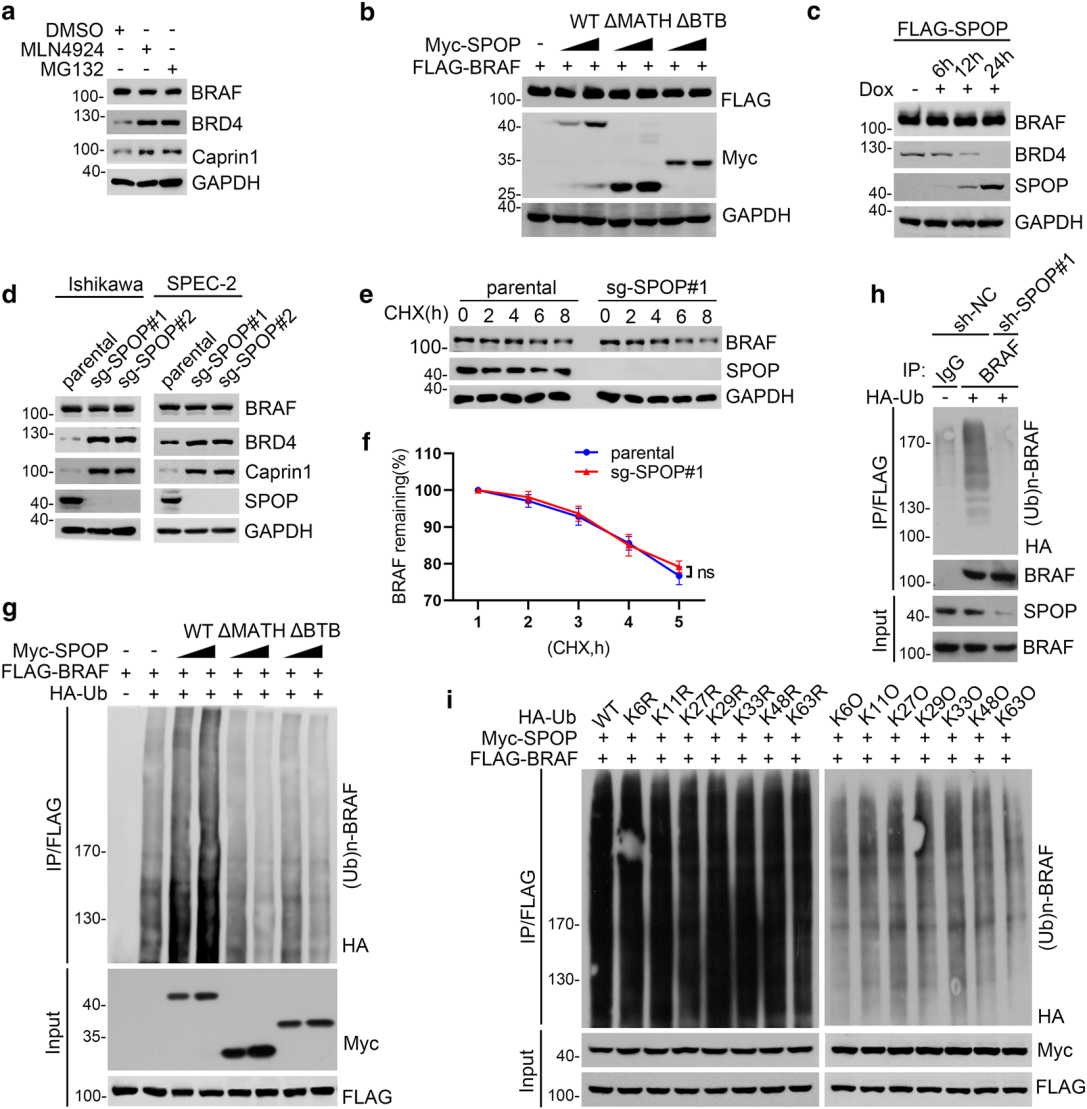
SPOP promotes non-degradative ubiquitination of BRAF. **a** Western blot of indicated proteins in WCL from Ishikawa cells treated with DMSO, MLN4924 (100 nM), or with MG132 (20 μM) for 8 h. **b** Western blot of WCL from 293 T cells transfected with FLAG-BRAF and increasing doses of Myc-tagged SPOP-WT or deletion mutants. **c** Western blot of indicated proteins in WCL from Tet-on-inducible Ishikawa cells treated with doxycycline (DOX). **d** Western blot of the indicated proteins in WCL from Ishikawa or SPEC-2 cells with SPOP knockout through CRISPR/Cas9 methods. **e**, **f** Western blot of indicated proteins in WCL from 293 T cells treated with 50 µg/ml cycloheximide (CHX) and harvested at different time points. Data were shown as mean ± SD (n = 3) **f**. The *p-values* were calculated using the Two-way ANOVA test. n.s., non-significant. **g**, **i** 293 T cells were transfected with the indicated plasmids. 24 h after transfection, the WCLs were prepared and the in vivo ubiquitination assays were performed. The polyubiquitinated forms of BRAF were detected by Western blot with anti-HA antibody. **h** 293 T cells were infected with lentivirus expressing SPOP-specific shRNA or negative control. The stable cell lines were transfection with HA-Ub and the cell lysates were prepared and immunoprecipitation was performed. The polyubiquitinated forms of BRAF were detected by Western blot with anti-HA antibody

Next, we investigated whether BRAF protein stability was controlled by the ubiquitin–proteasome pathway. Treatment of Ishikawa cells with the proteasome inhibitor MG132 had no obvious effect on BRAF protein levels, indicating that BRAF has a relative long half-life, at least in Ishikawa cells. MLN4924, a small-molecule inhibitor of the NEDD8-activating enzyme required for the activation of the CRL complex, did not alter BRAF protein levels. In contrast, MG132 or MLN4924 treatment caused a marked increase in the protein levels of BRD4 and Caprin1, which are two reported substrates of SPOP (Fig. 2a). Ectopic overexpression of SPOP-WT or its domain deletion mutants did not alter the levels of co-expressed BRAF (Fig. 2b). To characterize the effect of SPOP on endogenous BRAF, we generated Tet-on-inducible Ishikawa cells. Induction of FLAG-SPOP by doxycycline treatment did not affect BRAF protein levels, whereas BRD4 was destabilized in a time-dependent manner (Fig. 2c). Depletion of SPOP by shRNA-mediated knockdown or CRISPR/Cas9-mediated KO in EC cell lines elevated the protein levels of BRD4 and Caprin1, but not BRAF (Fig. 2d, Additional file 1: Fig. S1a–c). Moreover, the half-life of BRAF was comparable between parental and SPOP KO cells (Fig. 2e, f). Although BRAF was not degraded by SPOP, it was robustly polyubiquitinated in response to the co-expression of SPOP-WT but not by the SPOP-ΔBTB or -ΔMATH mutant (Fig. 2g). We further demonstrated that the SPOP–CUL3–RBX1 E3 ubiquitin ligase complex catalyzed BRAF polyubiquitination in vitro (Additional file 1: Fig. S1d). Accordingly, SPOP depletion decreased the ubiquitination levels of endogenous BRAF (Fig. 2h). These results indicate that SPOP induces non-degradative polyubiquitination of BRAF.

Given that SPOP-mediated BRAF ubiquitination is non-degradative, we examined the polyUb chain-linkage specificity of BRAF. We performed in vivo ubiquitination assays using a panel of ubiquitin mutants containing single KR mutations at each of the seven lysine residues. Co-expression of either Ub-KR mutant did not obviously alter SPOP-mediated BRAF ubiquitination. We also used a reciprocal series of Ub-KO mutants that contain only one lysine residue, with the other six lysine residues mutated to arginine. The expression of either Ub-KO mutant nearly abolished SPOP-mediated BRAF ubiquitination (Fig. 2i), indicating that SPOP may catalyze the synthesis of mixed-linkage polyUb chains on BRAF. We also utilized linkage-specific K27/K29/48/63-Ub antibodies to demonstrate that ubiquitinated BRAF contains K27-, K29-, K48-, and K63-Ub linkages (Additional file 1: Fig. S1e). Taken together, our data indicate that SPOP promotes non-degradative ubiquitination of BRAF.

### The SBC motif in BRAF is required for SPOP-mediated BRAF ubiquitination

**Fig. 3:**
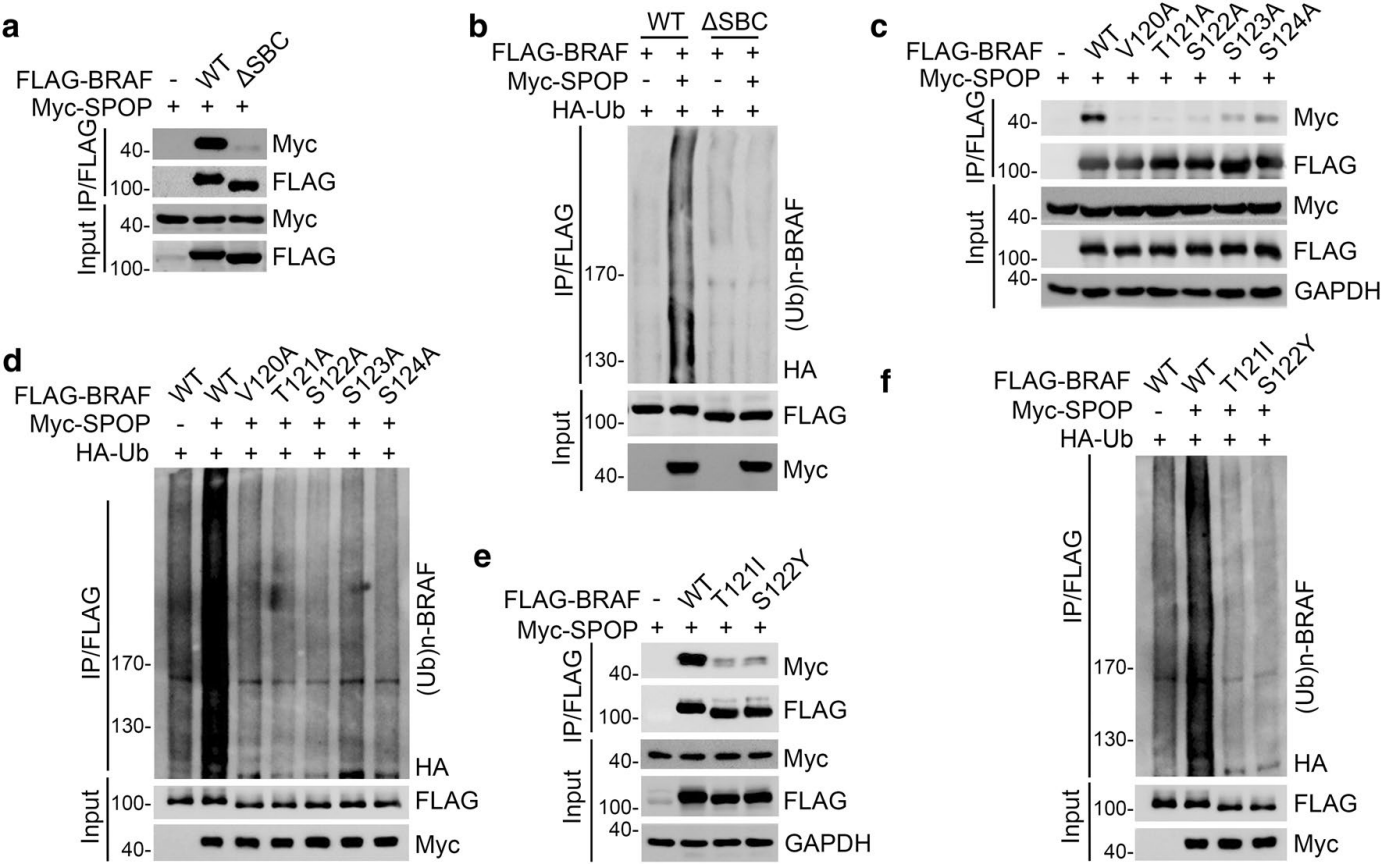
The SBC motif in BRAF is recognized by SPOP. **a**, **c**, **e** Western blot of WCL and co-IP samples of anti-FLAG antibody obtained from 293 T cells transfected with BRAF-WT or deletion mutants. **b**, **d**, **f** 293 T cells were transfected with the indicated plasmids. 24 h after transfection, the WCLs were prepared and the in vivo ubiquitination assays were performed. The polyubiquitinated forms of BRAF were detected by Western blot with anti-HA antibody

To examine whether the potential SBC motif is required for the SPOP-BRAF interaction, we generated a BRAF mutant in which the SBC motif was deleted. Co-IP assay results showed that SPOP only interacted with wild-type BRAF but did not bind to the BRAF-ΔSBC mutant (Fig. 3a, Additional file 1: Fig. S2a). The deletion of the SBC motif in BRAF abrogated SPOP-mediated BRAF ubiquitination (Fig. 3b). Mutations in amino acids in the SBC motif considerably reduced the SPOP-BRAF interaction (Fig. 3c) and SPOP-mediated BRAF ubiquitination (Fig. 3d).

Given that BRAF interacts with SPOP in an SBC motif-dependent manner, we further explored whether cancer-associated BRAF mutations that occur in the SBC motif would attenuate the SPOP-BRAF interaction and permit BRAF to evade SPOP-mediated ubiquitination. To this end, we examined the cancer sequencing data deposited in cancer gene mutation databases (cBioPortal and COSMIC). We identified a BRAF-T121I mutation in glioblastoma and BRAF-S122Y mutation in osteosarcoma (Fig. 1b, Additional file 1: Table. S3). We found that the SPOP-binding capacity of these BRAF mutants was markedly reduced (Fig. 3e, Additional file 1: Fig. S2b), and SPOP-mediated ubiquitination of these mutants was also markedly attenuated (Fig. 3f). Taken together, our data indicate that cancer-derived BRAF mutations occurring in the SBC motif allow BRAF to evade SPOP-mediated ubiquitination.

### EC- and PCa-associated SPOP mutants are defective in promoting BRAF ubiquitination

**Fig. 4:**
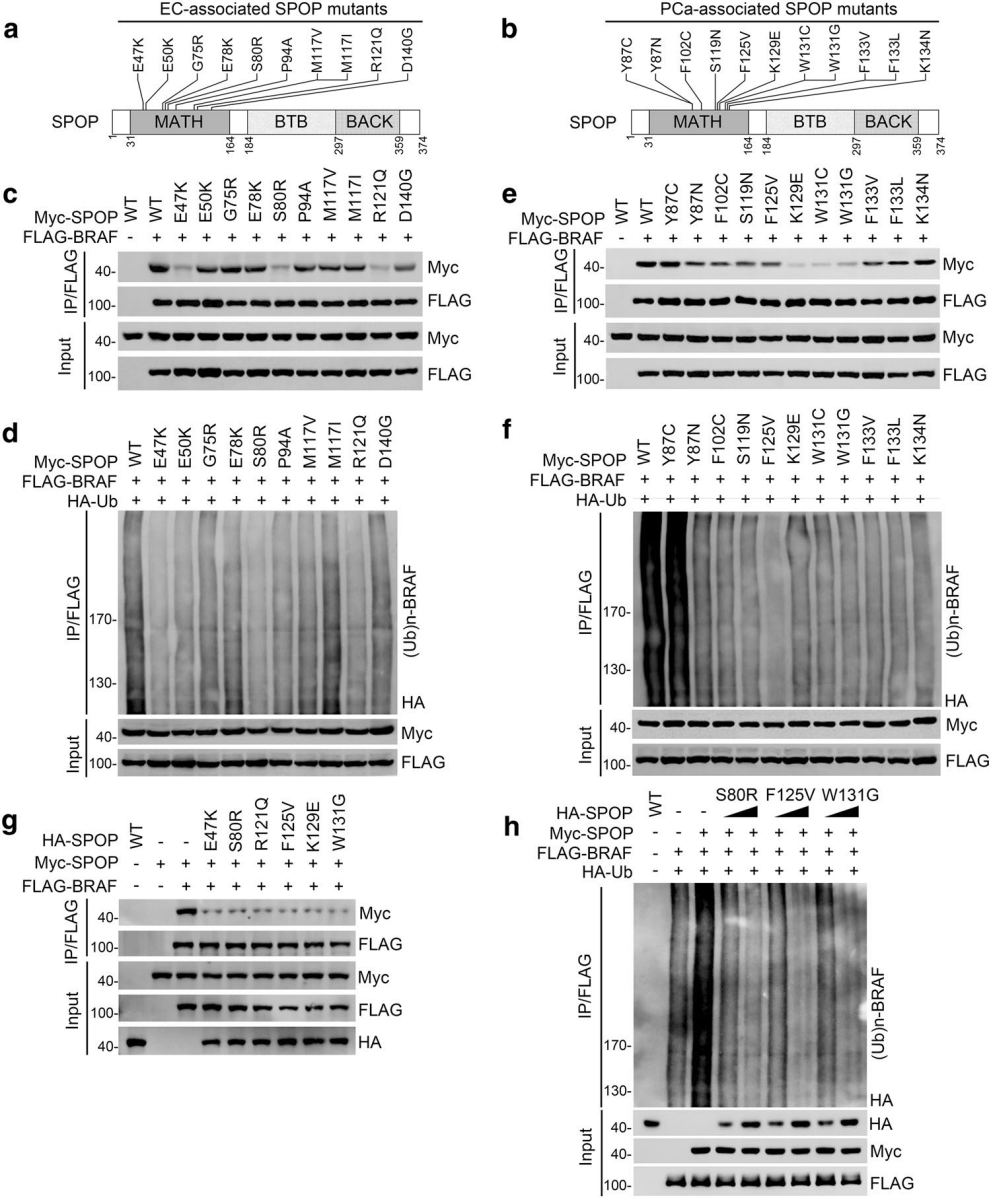
EC- or PCa-associated SPOP mutants are defective in promoting BRAF ubiquitination. **a**, **b** Distribution of the recurrent mutation sites on the SPOP found in EC(a) or PCa **b**. **c**, **e**, **g** Western blot of WCL and co-IP samples of anti-FLAG antibody obtained from (293 T cells transfected with the indicated plasmids. **d**, **f**, **h** 293 T cells were transfected with the indicated plasmids. The WCLs were prepared and the in vivo ubiquitination assays were performed. The polyubiquitinated forms of BRAF were detected by Western blot with anti-HA antibody

To date, the vast majority of SPOP mutations associated with EC or PCa occur within the MATH domain, which is responsible for selective substrate binding (Fig. 4a, b) [10, 11]. We postulated that EC- or PCa-associated SPOP mutants may be defective in mediating BRAF ubiquitination. The BRAF-binding ability of EC-associated SPOP mutants was moderately or severely impaired as compared to that of SPOP-WT (Fig. 4c). SPOP-mediated BRAF ubiquitination was moderately or severely attenuated in these mutants (Fig. 4d). Similar effects were observed when PCa-associated SPOP mutants were tested (Fig. 4e, f). In accordance with a previous study showing that cancer-associated SPOP mutants function as dominant-negative variants to deregulate their substrates [12], we found that co-expression of the SPOP mutant markedly reduced the interaction between SPOP-WT and BRAF (Fig. 4g), resulting in the suppression of wild-type SPOP-mediated BRAF ubiquitination (Fig. 4h). Taken together, our data indicate that EC or PCa-associated SPOP mutants exert dominant-negative effects by downregulating BRAF ubiquitination due to impaired BRAF binding capacity.

### Cytoplasmic but not nuclear SPOP promotes BRAF ubiquitination

**Fig. 5:**
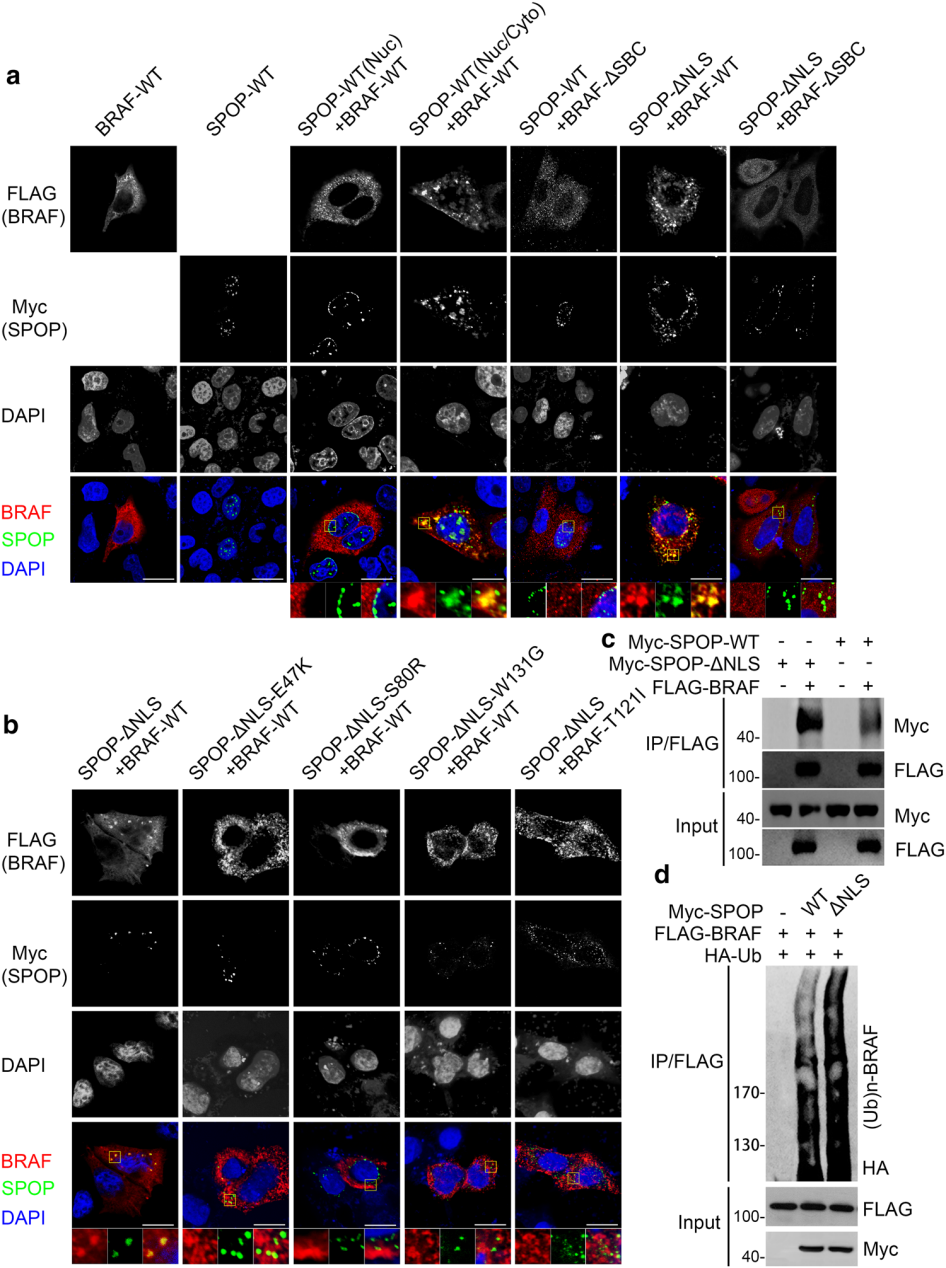
The SPOP-BRAF interaction occurs in the cytoplasm. **a**, **b** Representative images of HeLa cells transfected with indicated plasmids, and then immunostained with Myc (SPOP), FLAG (BRAF), and DAPI. Scale bar, 10 μm. Enlargements of selected areas were shown. **c** Western blot of the indicated proteins in WCL and samples from co-IP with anti-FLAG antibody in 293 T cells transfected with the indicated plasmids. **d** 293 T cells were transfected with the indicated plasmids. The WCLs were prepared and the in vivo ubiquitination assays were performed. The polyubiquitinated forms of BRAF were detected by Western blot with anti-HA antibody

BRAF is a cytoplasmic protein kinase. Our previous study showed that SPOP is localized exclusively in the nucleus as speckles or in both the cytoplasm and nucleus [19]. We investigated the subcellular localization of the SPOP-BRAF interaction. BRAF was diffusely localized in the cytoplasm. When SPOP was localized in both the cytoplasm and nucleus, BRAF was recruited into SPOP speckles in the cytoplasm. When SPOP was localized exclusively in the nucleus, it did not co-localize with cytoplasmic BRAF. We found that SPOP lacking the NLS sequence (SPOP-ΔNLS) accumulated exclusively in the cytoplasm in punctate patterns and colocalized perfectly with cytoplasmic BRAF. Moreover, the deletion of the SBC motif in BRAF did not alter its diffuse cytoplasmic localization, and the SPOP-induced speckle pattern of BRAF was not observed (Fig. 5a).

Next, we investigated the effect of cancer-associated SPOP mutations on BRAF localization. We focused on three hotspot mutants (E47K, S80R, and W131G). Three cancer-associated SPOP mutants lacking the NLS sequence (ΔNLS-E47K, -S80R, and -W131G) accumulated exclusively in the cytoplasm in punctate patterns similar to those of SPOP-ΔNLS, but these mutants did not colocalize with BRAF. Similarly, the cancer-associated BRAF-T121I mutant did not co-localize with SPOP (Fig. 5b). Our previous studies showed that the SPOP-ΔNLS mutant was more effective in promoting the ubiquitination of its cytoplasmic substrates (INF2, MyD88, Caprin1, and HIPK2) than SPOP-WT, but it was unable to ubiquitinate nuclear substrates [19–22]. Indeed, we found that SPOP-ΔNLS immunoprecipitated more BRAF than SPOP-WT (Fig. 5c) and SPOP-ΔNLS was more effective in promoting BRAF ubiquitination than SPOP-WT (Fig. 5d). Taken together, our data indicate that SPOP can recruit BRAF into cytoplasmic speckles and that this activity strictly depends on its cytoplasmic localization.

### SPOP suppresses MAPK/ERK pathway activation

**Fig. 6:**
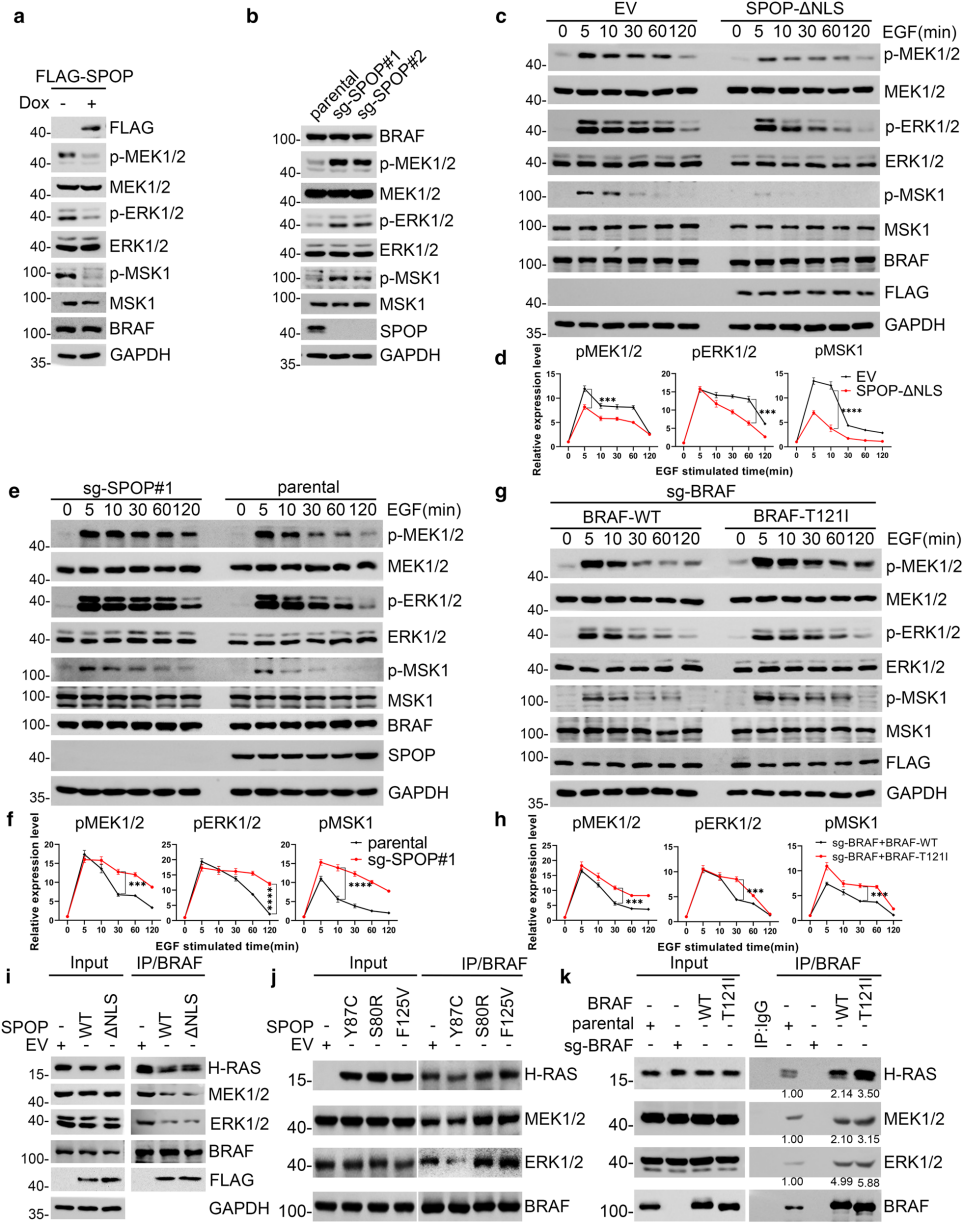
SPOP suppresses the activation of MAPK/ERK cascade. **a** Western blot of indicated proteins in WCLs from Tet-on-inducible Ishikawa cells treated with DOX. **b** Western blot of the indicated proteins in WCLs from parental and SPOP-KO Ishikawa cells. **c**, **d** EV and SPOP-ΔNLS overexpressed Ishikawa cells were serum-starved for 48 h and then treated with EGF (100 nM) for the corresponding times. The WCLs were prepared for WB analysis. At each time point, the intensity of p-ERK1/2, p-MEK1/2, p-MSK1 was normalized to the intensity of ERK1/2, MEK1/2, MSK1 respectively and then to the value at 0 min **d**. **e**, **f** Parental and SPOP-KO Ishikawa cells were serum-starved for 48 h and then treated with EGF (100 nM) for the corresponding times. The WCLs were prepared for WB analysis. At each time point, the intensity of p-ERK1/2, p-MEK1/2, p-MSK1 was normalized to the intensity of ERK1/2, MEK1/2, MSK1 respectively and then to the value at 0 min **f**. **g**, **h** SPOP-KO Ishikawa cells stably overexpressing BRAF-WT, or T121I mutant were serum-starved for 48 h and then treated with EGF (100 nM) for the corresponding times. The WCLs were prepared for WB analysis. At each time point, the intensity of p-ERK1/2, p-MEK1/2, p-MSK1 was normalized to the intensity of ERK1/2, MEK1/2, MSK1 respectively and then to the value at 0 min **h**. **i**, **j** The WCLs from SPOP-KO Ishikawa cells stably overexpressing EV, SPOP-WT, SPOP- ΔNLS **i** and mutants **j** were prepared and subjected to co-IP with anti-BRAF antibody. The immunoprecipitates were analyzed by WB with the indicated antibodies. **k** The WCLs from BRAF-KO Ishikawa cells stably overexpressing BRAF-WT or T121I mutant were prepared and subjected to co-IP with anti-BRAF antibody. The immunoprecipitates were analyzed by WB with the indicated antibodies. The band intensity of the indicated proteins was first normalized to loading input l and the value was further normalized to the one in cells. All data shown are mean values ± SD (n = 3). The *p-values* were calculated using the Two-way ANOVA test in **c**, **e**, **g**. *p < 0.05, **p < 0.01, ***p < 0.001, ****p < 0.0001

BRAF plays a critical role in the regulation of MAPK/ERK pathway activation [3]. We examined MAPK/ERK activity in SPOP KO and SPOP overexpressed cells. The induction of FLAG-SPOP by doxycycline treatment led to a marked decrease in the phosphorylation levels of MEK1/2, ERK1/2, and MSK1, indicating that MAPK/ERK activation was attenuated (Fig. 6a). Conversely, stronger MAPK/ERK activation was observed when SPOP was ablated (Fig. 6b). Moreover, SPOP-ΔNLS overexpression attenuated the MAPK/ERK pathway activation in EGF-stimulated cells (Fig. 6c, d). Conversely, SPOP KO enhanced MAPK/ERK pathway activation in EGF-stimulated cells (Fig. 6e, f). Stronger MAPK/ERK activation was observed in SPOP KO Ishikawa cells reconstituted with the SPOP-S80R mutant than in those reconstituted with the SPOP-Y87C mutant, which retains a similar binding capacity to BRAF as SPOP-WT (Additional file 1: Fig. S3a, b).

To determine whether the escape of SPOP-mediated-BRAF ubiquitination has any impact on MAPK/ERK pathway activation, we reconstituted BRAF-KO Ishikawa cells (Additional file 1: Fig. S3c–e) with BRAF-WT or the -T121I mutant, which is incapable of binding to SPOP. As shown in Fig. 6g, h, EGF-induced MAPK/ERK activation was stronger in BRAF-KO Ishikawa cells reconstituted with the BRAF-T121 mutant than in those reconstituted with BRAF-WT. We found that stable overexpression of SPOP-WT or ΔNLS mutant reduced the interaction between BRAF and its upstream regulator, H-RAS, and downstream effectors MEK1/2 and ERK1/2 (Fig. 6i). Conversely, stable overexpression of SPOP-S80R or -F125V, increased the interactions between BRAF and MAPK/ERK pathway components. Surprisingly, stable overexpression of the SPOP-Y87C mutant even moderately reduced the interactions between BRAF and MAPK/ERK pathway components (Fig. 6j). Moreover, the interactions between BRAF and MAPK/ERK pathway components were moderately increased in BRAF-KO Ishikawa cells reconstituted with the BRAF-T121 mutant as compared to those reconstituted with BRAF-WT (Fig. 6k). Taken together, our data indicate that SPOP suppresses MAPK/ERK pathway activation.

### BRAF-T121I mutant is more effective to promote cell growth, migration, and invasion than BRAF-WT

**Fig. 7:**
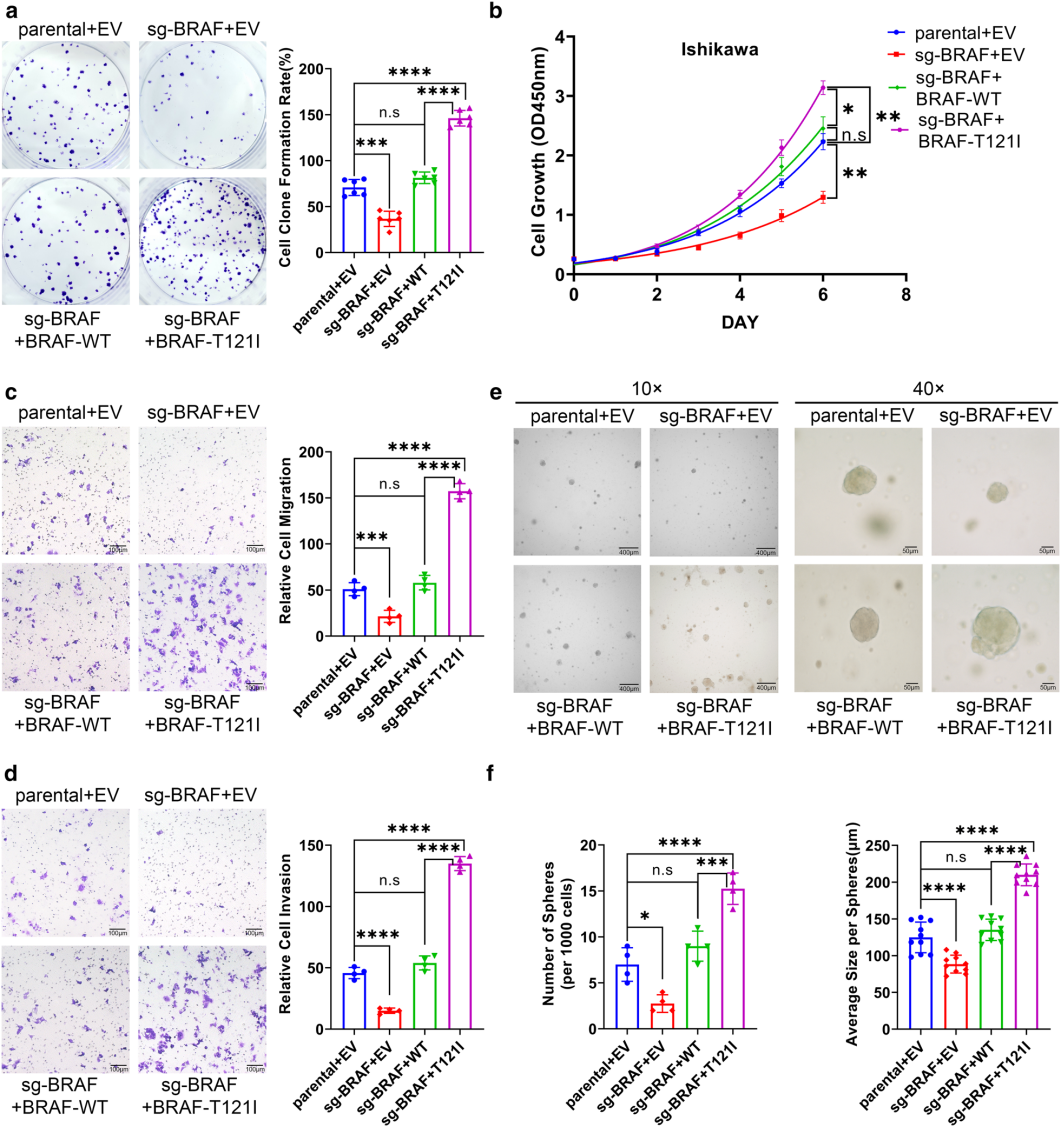
Deficiency in SPOP-mediated BRAF ubiquitination confers oncogenicity. **a** Clone formation assays in parental, BRAF-KO Ishikawa cells, or BRAF-KO Ishikawa cells stably overexpressing BRAF-WT or BRAF-T121I mutant. Quantitative data are shown in the right panel. Data are shown as the means ± SD (n = 4). **b** CCK-8 assays in parental, BRAF-KO Ishikawa cells, or BRAF-KO Ishikawa cells stably overexpressing BRAF-WT or BRAF-T121I mutant. Data are shown as the means ± SD (n = 4). **c** Transwell migration assays in parental, BRAF-KO Ishikawa cells, or BRAF-KO Ishikawa cells stably overexpressing BRAF-WT or BRAF-T121I mutant. Quantitative data are shown in the right panel. Data are shown as the means ± SD (n = 4). **d** Transwell invasion assays in parental, BRAF-KO Ishikawa cells, or BRAF-KO Ishikawa cells stably overexpressing BRAF-WT or BRAF-T121I mutant. Quantitative data are shown in the right panel. Data are shown as the means ± SD (n = 4). **e** Representative pictures of 3D sphere formation assays in parental, BRAF-KO Ishikawa cells, or BRAF-KO Ishikawa cells stably overexpressing BRAF-WT or BRAF-T121I mutant. Scale bars, 10 × image: 400 μm, 40 × image: 50 μm. **f** Quantitative data of parental, BRAF KO and rescued with BRAF or BRAF mutation in BRAF-knock-out Ishikawa cells after 10 days in three-dimensional culture. Numbers and average sizes per sphere. Data are shown as means ± SD (n = 4 in left, n = 10 in right). The *p-values* were calculated using the One-way ANOVA test in **a**, **c**, **d**, **f** and the Two-way ANOVA test in **b**. *p < 0.05, **p < 0.01, ***p < 0.001, ****p < 0.0001

We found that stable overexpression of SPOP-WT reduced the growth of Ishikawa, KLE, and SPEC-2 cells as compared to parental cells. In contrast, stable overexpression of EC-associated SPOP mutants increased the growth of KLE, SPEC-2, and Ishikawa cells as compared to parental cells (Additional file 1: Fig. S4a–d). Consistent with the substantial roles of BRAF in cellular malignancy [5], BRAF KO Ishikawa cells grew at a markedly reduced growth rate, as demonstrated by colony formation and CCK-8 assays. Reconstitution with BRAF-WT restored BRAF-KO cell growth in vitro, and more importantly, BRAF-KO cells reconstituted with the BRAF-T121I mutant showed a higher growth rate than those reconstituted with BRAF-WT (Fig. 7a, b). Similarly, Transwell assay results showed that BRAF-T121I mutant expression resulted in increased cell migration and invasion compared to BRAF-WT (Fig. 7c, d). Moreover, the 3D sphere formation assay results showed that the number and size of spheres in BRAF KO cells reconstituted with the BRAF-T121I mutant were markedly increased compared to those reconstituted with BRAF-WT, suggesting that the cancer-derived BRAF mutant increased anchorage-independent cell growth (Fig. 7e, f). Taken together, our data indicate that loss of SPOP-mediated BRAF ubiquitination promotes cell malignancy.

## Discussion

**Fig. 8:**
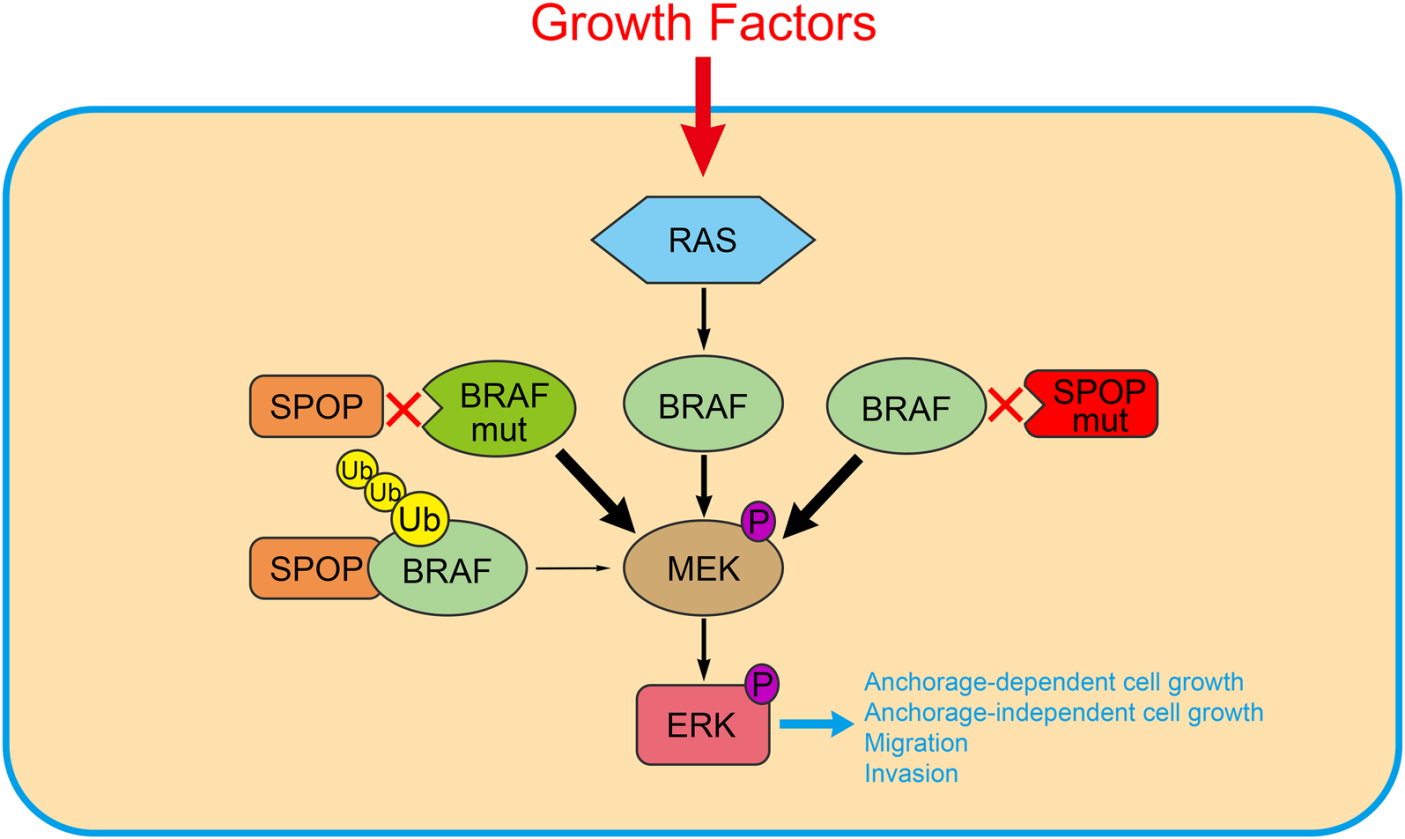
A model was proposed according to the findings of the present study. The schematic diagram depicts a model that SPOP regulates the MAPK/ERK pathway through promoting BRAF ubiquitination

Aberrant activation of the MAPK/ERK cascade is common in human cancer. BRAF mutations are frequently observed in melanoma, thyroid cancer, and others. In contrast, oncogenic BRAF mutations are uncommon in EC and PCa, with prevalence rates ranging from 2 to 5%, depending on the specific case series [23, 24]. Whether specific genetic alterations drive aberrant regulation of wild-type BRAF activity in EC and PCa remains poorly understood. In the current study, we showed that the CRL3-SPOP complex mediates the non-degradative ubiquitination of BRAF, which reduces the interaction between BRAF and other MAPK/ERK pathway components, resulting in subsequent MAPK/ERK inactivation (Fig. 8). Importantly, EC- or PCa-associated SPOP mutants showed reduced capacity to ubiquitinate BRAF. Moreover, some cancer-associated BRAF mutants (T121I and S122Y) evaded SPOP-mediated regulation and were stronger activators of the MAPK/ERK cascade than wild-type BRAF. We noted that the interactions between BRAF and the MAPK/ERK pathway components were only moderately increased in BRAF-T121 reconstituted cells to BRAF-WT-reconstituted cells. It would be interesting to investigate whether this effect is more obvious in growth factor-treated conditions. Taken together, our data raise the possibility that disruption of the SPOP-BRAF regulatory axis may promote malignant transformation of cancer cells.

Proteins can be tagged with mono-ubiquitin or modified by polyUb chains that can vary in length and linkage specificity, and these variations influence how ubiquitination signals are interpreted [25]. Accumulating evidence suggests that both degradative and nondegradative ubiquitination play vital roles in modulating BRAF protein stability and function. RNF149, FBXW7, and TRAF2 have been reported as E3 ubiquitin ligases of BRAF [26–28]. USP28, a deubiquitinating enzyme, causes BRAF degradation by stabilizing FBXW7 [29]. In melanoma cells treated with proinflammatory cytokines, the HECT domain-containing E3 ligase ITCH catalyzes the non-degradative K27-linked ubiquitination of BRAF, which leads to PP2A recruitment, 14-3-3 dissociation, and sustained BRAF/MEK/ERK pathway activation [30]. This effect is distinct from our finding that SPOP-mediated BRAF ubiquitination inactivates BRAF. One possible explanation for this discrepancy could be that the different linkage types of polyUb chains catalyzed by CRL3-SPOP or ITCH determine the distinct fates of BRAF. Whether PolyUb chains on BRAF catalyzed by CRL3-SPOP directly hinder BRAF complex assembly or recruit other ubiquitin-binding proteins should be investigated in future studies.

The CRL3-SPOP complex catalyzes both degradative and non-degradative ubiquitination depending on the substrate. For example, CRL3-SPOP catalyzes K27-linked non-degradative ubiquitination of Geminin, which prevents DNA replication over-firing by indirectly blocking the association of Cdt1 with the MCM protein complex [31]. CRL3-SPOP catalyzed mixed K6-, K27-, and K29-linked non-degradative ubiquitination of p62, which suppresses p62-dependent autophagy [32]. CRL3-SPOP catalyzes mixed K27-, K29-, K33-, K48-, and K63-linked non-degradative ubiquitination of INF2 and HIPK2 to regulate mitochondrial division and DNA damage response, respectively [19, 22]. In the current study, we found that SPOP-mediated BRAF ubiquitination had no predominant ubiquitination linkage specificity, in sharp contrast to other SPOP substrates reported using Ub linkage mutants. However, the mechanisms underlying this phenomenon are currently unknown. Given that Ub linkage mutants have been artificially generated, non-physiological effects may occur. Alternatively, we used a panel of linkage-specific Ub antibodies to demonstrate that ubiquitinated BRAF at least contains K27-, K29-, K48-, and K63-Ub linkages. Further studies are needed to thoroughly clarify the polyUb-linkage composition using mass spectrometry-based proteomics, such as the selected reaction monitoring (Ub-AQUA-SRM) method [33].

Targeted treatment with BRAF and MEK inhibition has demonstrated clinical efficacy in melanomas harboring the BRAF-V600E/K mutation, and the combination of dabrafenib and trametinib, vemurafenib, and cobimetinib represent effective therapeutic options for these patients [5]. However, vemurafenib and dabrafenib caused a paradoxical induction of MAPK/ERK pathway activation and induced proliferation via a RAS-dependent mechanism in wild-type BRAF cells [34]. AZ304, a potent dual BRAF inhibitor of both wild-type and BRAF-V600E mutant BRAF, exerts anti-tumor effects in colorectal cancer independent of BRAF genetic status [35]. Given that BRAF activity is elevated in SPOP-mutated cancers, the efficacy of AZ304 in these cancers should be investigated.

## Conclusions

Our findings show that SPOP and BRAF are biochemically linked. SPOP promoted non-degradative ubiquitination of BRAF, which resulted in subsequent MAPK/ERK inactivation. Importantly, PCa- or EC-associated SPOP mutations lead to abnormal activation of the MAPK/ERK pathway in a BRAF-dependent manner. Our data raise the possibility that disruption of the SPOP-BRAF regulatory axis may promote the malignant transformation of cancer cells (Fig. 8). Given that BRAF activity is elevated in SPOP-mutated cancers, elucidation of the SPOP-BRAF regulatory axis in further studies will be helpful in designing therapeutic strategies for SPOP-mutated cancers.

## Supplementary Information

**Additional file 1: Figure S1.** SPOP promotes non-degradative ubiquitination of BRAF (related to Figure 2). (a) Western blot of the indicated proteins in WCL from Ishikawa and KLE cells infected with lentivirus expressing SPOP-specific shRNAs or negative control. (b) Schematic of CRISPR/Cas9-mediated knockout of SPOP by sgRNA#1 or sgRNA#2 in Ishikawa cells. (c) Sanger sequencing confirming that the SPOP gene was edited by sgRNA#1 or sgRNA#2 in Ishikawa cells. (d) Western blot of the products of *in vitro* ubiquitination assays performed by incubating the reconstituted SPOP–CUL3–RBX1 E3 ligase complex with E1 and E2 enzymes, ubiquitin and GST-BRAF at 30 °C for 2 h. (e) Western blot of the products of *in vivo *ubiquitination assays from 293T cells transfected with the indicated plasmids. **Figure S2.** The SBC motif in BRAF is recognized by SPOP (related to Figure 3). (a, b) Western blot of WCL and co-IP samples of anti-FLAG antibody obtained from 293T cells transfected with the indicated plasmids. **Figure S3.** SPOP suppresses the activation of MAPK/ERK cascade (related to Figure 6). (a, b) SPOP-KO Ishikawa cells stably overexpressing SPOP-Y87C, or S80R mutant were serum-starved for 48 hr and then treated with EGF (100 nM) for the corresponding times. The WCLs were prepared for WB analysis. At each time point, the intensity of p-ERK1/2, p-MEK1/2, p-MSK1 was normalized to the intensity of ERK1/2, MEK1/2, MSK1 respectively and then to the value at 0 min (b). (c) Schematic of CRISPR/Cas9-mediated knockout of BRAF by sgRNA in Ishikawa cells. (d) Sanger sequencing confirming that the BRAF gene was edited by sgRNA in Ishikawa cells. (e) Western blot of the indicated proteins in WCLs from parental and BRAF-KO Ishikawa cells. All data shown are mean values ± SD(n=3). The *p-values* were calculated using the Two-way ANOVA test in (b). *p<0.05, **p<0.01, ***p<0.001, ****p<0.0001. **Figure S4.** The impact of SPOP-WT and EC-associated SPOP mutants on the growth of three EC cell lines. (a-c) CCK-8 assays in KLE (a), SPEC-2 (b), and Ishikawa (c) cells stably overexpressing EV, SPOP-WT or EC-associated SPOP mutants. Data are shown as the means ± SD (n=4). The *p-values* were calculated using the Two-way ANOVA test in (a-c). *p<0.05, **p<0.01, ***p<0.001, ****p<0.0001. (d) Western blot of the indicated proteins in WCL from KLE, SPEC-2, and Ishikawa cells stably overexpressing EV, SPOP-WT, or EC-associated SPOP mutants. **Table S1.** Sequence information. **Table S2.** Antibody and recombinant protein information. **Table S3.** Cell cultures, chemicals and Kit.

## Data Availability

All data generated or analyzed in this study are included in this published article. All the data supporting the findings of this study are available from the corresponding author upon request.
