# Supplementary material for: SPOP inhibits BRAF-dependent tumorigenesis through promoting non-degradative ubiquitination of BRAF
Source: Cell Biosci. 2022 Dec 30;12:211. doi: 10.1186/s13578-022-00950-z (PMC9805134; doi:10.1186/s13578-022-00950-z)
Supplement: Supplementary file 1 — Additional file 1: Figure S1. SPOP promotes non-degradative ubiquitination of BRAF (related to Figure 2). (a) Western blot of the indicated proteins in WCL from Ishikawa and KLE cells infected with lentivirus expressing SPOP-specific shRNAs or negative control. (b) Schematic of CRISPR/Cas9-mediated knockout of SPOP by sgRNA#1 or sgRNA#2 in Ishikawa cells. (c) Sanger sequencing confirming that the SPOP gene was edited by sgRNA#1 or sgRNA#2 in Ishikawa cells. (d) Western blot of the products of in vitro ubiquitination assays performed by incubating the reconstituted SPOP–CUL3–RBX1 E3 ligase complex with E1 and E2 enzymes, ubiquitin and GST-BRAF at 30 °C for 2 h. (e) Western blot of the products of in vivo ubiquitination assays from 293T cells transfected with the indicated plasmids. Figure S2. The SBC motif in BRAF is recognized by SPOP (related to Figure 3). (a, b) Western blot of WCL and co-IP samples of anti-FLAG antibody obtained from 293T cells transfected with the indicated plasmids. Figure S3. SPOP suppresses the activation of MAPK/ERK cascade (related to Figure 6). (a, b) SPOP-KO Ishikawa cells stably overexpressing SPOP-Y87C, or S80R mutant were serum-starved for 48 hr and then treated with EGF (100 nM) for the corresponding times. The WCLs were prepared for WB analysis. At each time point, the intensity of p-ERK1/2, p-MEK1/2, p-MSK1 was normalized to the intensity of ERK1/2, MEK1/2, MSK1 respectively and then to the value at 0 min (b). (c) Schematic of CRISPR/Cas9-mediated knockout of BRAF by sgRNA in Ishikawa cells. (d) Sanger sequencing confirming that the BRAF gene was edited by sgRNA in Ishikawa cells. (e) Western blot of the indicated proteins in WCLs from parental and BRAF-KO Ishikawa cells. All data shown are mean values ± SD(n=3). The p-values were calculated using the Two-way ANOVA test in (b). *p<0.05, **p<0.01, ***p<0.001, ****p<0.0001. Figure S4. The impact of SPOP-WT and EC-associated SPOP mutants on the growth of three EC cell lines. (a-c) CCK- [file 13578_2022_950_MOESM1_ESM.docx]

Additional file for

**SPOP inhibits BRAF-dependent tumorigenesis through promoting non-degradative ubiquitination of BRAF**

Kai Feng, Qing Shi, Dongyue Jiao, Yingji Chen, Wanqi Yang, Ke Su, Yalan Wang, Yan Huang, Pingzhao Zhang, Yao Li, and Chenji Wang


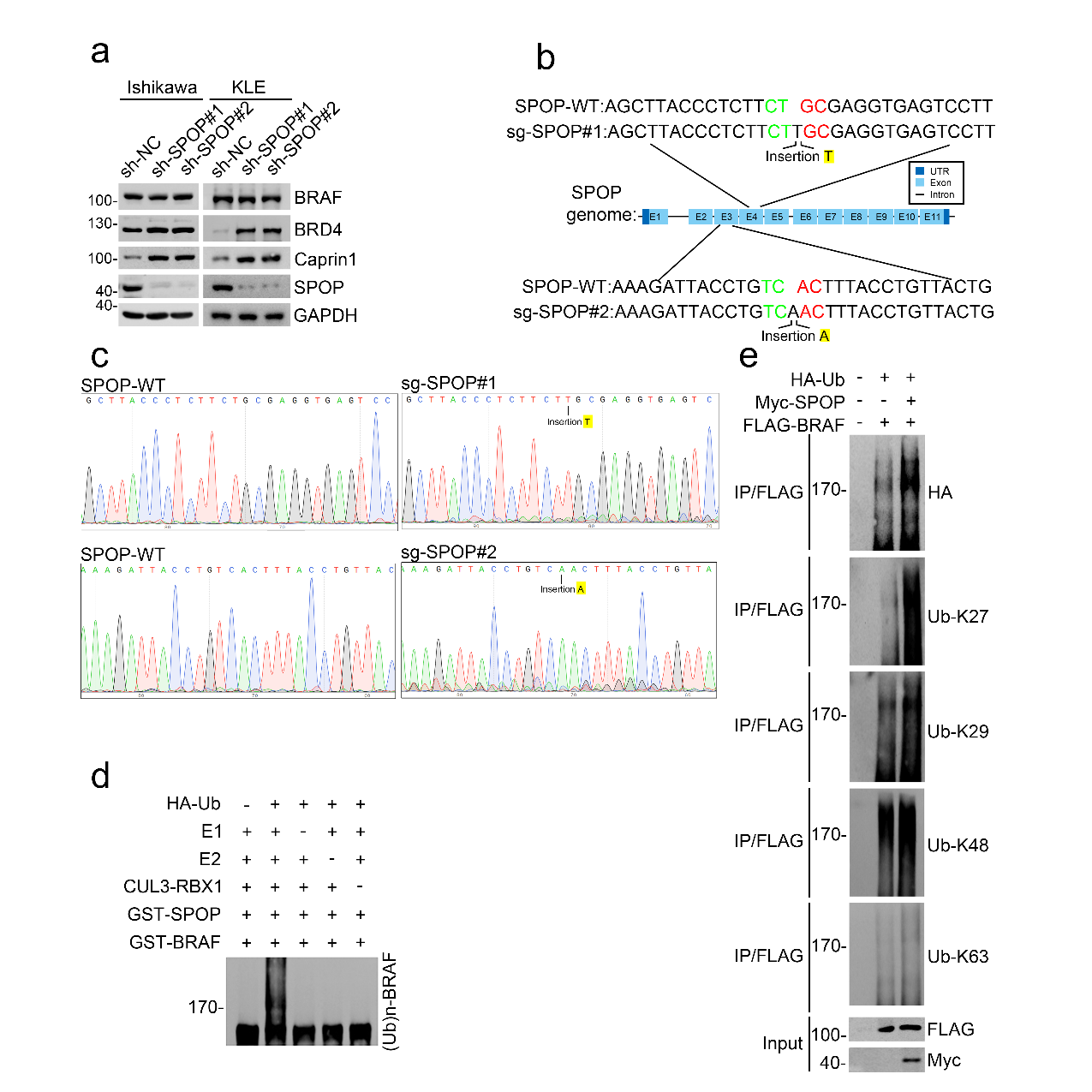


**Figure S1. SPOP promotes non-degradative ubiquitination of BRAF (related to Figure 2).**

(a) Western blot of the indicated proteins in WCL from Ishikawa and KLE cells infected with lentivirus expressing SPOP-specific shRNAs or negative control.

(b) Schematic of CRISPR/Cas9-mediated knockout of SPOP by sgRNA#1 or sgRNA#2 in Ishikawa cells.

(c) Sanger sequencing confirming that the SPOP gene was edited by sgRNA#1 or sgRNA#2 in Ishikawa cells.

(d) Western blot of the products of *in vitro* ubiquitination assays performed by incubating the reconstituted SPOP–CUL3–RBX1 E3 ligase complex with E1 and E2 enzymes, ubiquitin and GST-BRAF at 30 °C for 2 h.

(e) Western blot of the products of *in vivo* ubiquitination assays from 293T cells transfected with the indicated plasmids.


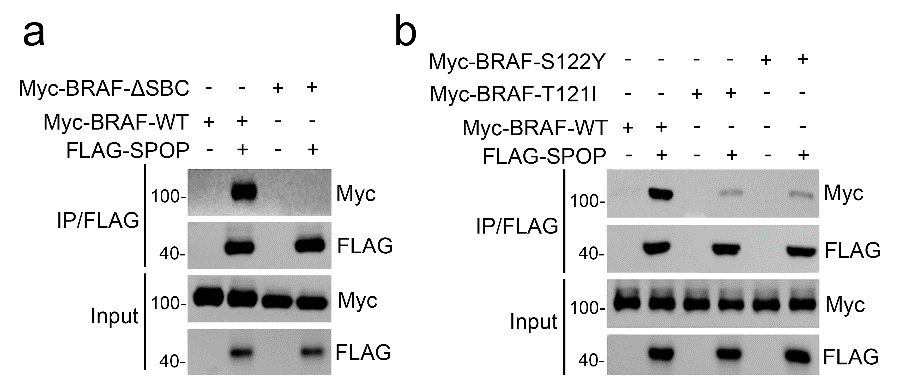


**Figure S2. The SBC motif in BRAF is recognized by SPOP (related to Figure 3).**

(a, b) Western blot of WCL and co-IP samples of anti-FLAG antibody obtained from 293T cells transfected with the indicated plasmids.


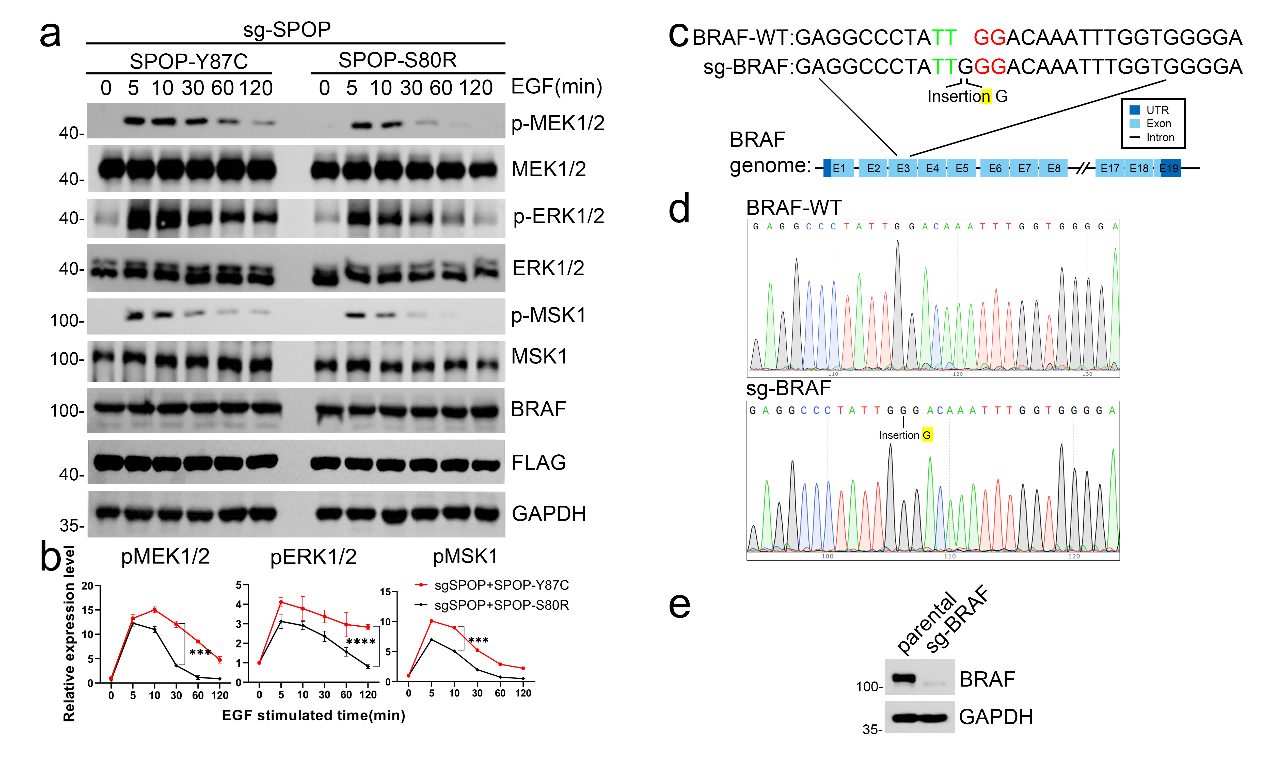
**Figure S3. SPOP suppresses the activation of MAPK/ERK cascade (related to Figure 6).**

(a, b) SPOP-KO Ishikawa cells stably overexpressing SPOP-Y87C, or S80R mutant were serum-starved for 48 hr and then treated with EGF (100 nM) for the corresponding times. The WCLs were prepared for WB analysis. At each time point, the intensity of p-ERK1/2, p-MEK1/2, p-MSK1 was normalized to the intensity of ERK1/2, MEK1/2, MSK1 respectively and then to the value at 0 min (b).

(c) Schematic of CRISPR/Cas9-mediated knockout of BRAF by sgRNA in Ishikawa cells.

(d) Sanger sequencing confirming that the BRAF gene was edited by sgRNA in Ishikawa cells.

(e) Western blot of the indicated proteins in WCLs from parental and BRAF-KO Ishikawa cells.

All data shown are mean values ± SD(n=3). The *p-values* were calculated using the Two-way ANOVA test in (b). *p<0.05, **p<0.01, ***p<0.001, ****p<0.0001.


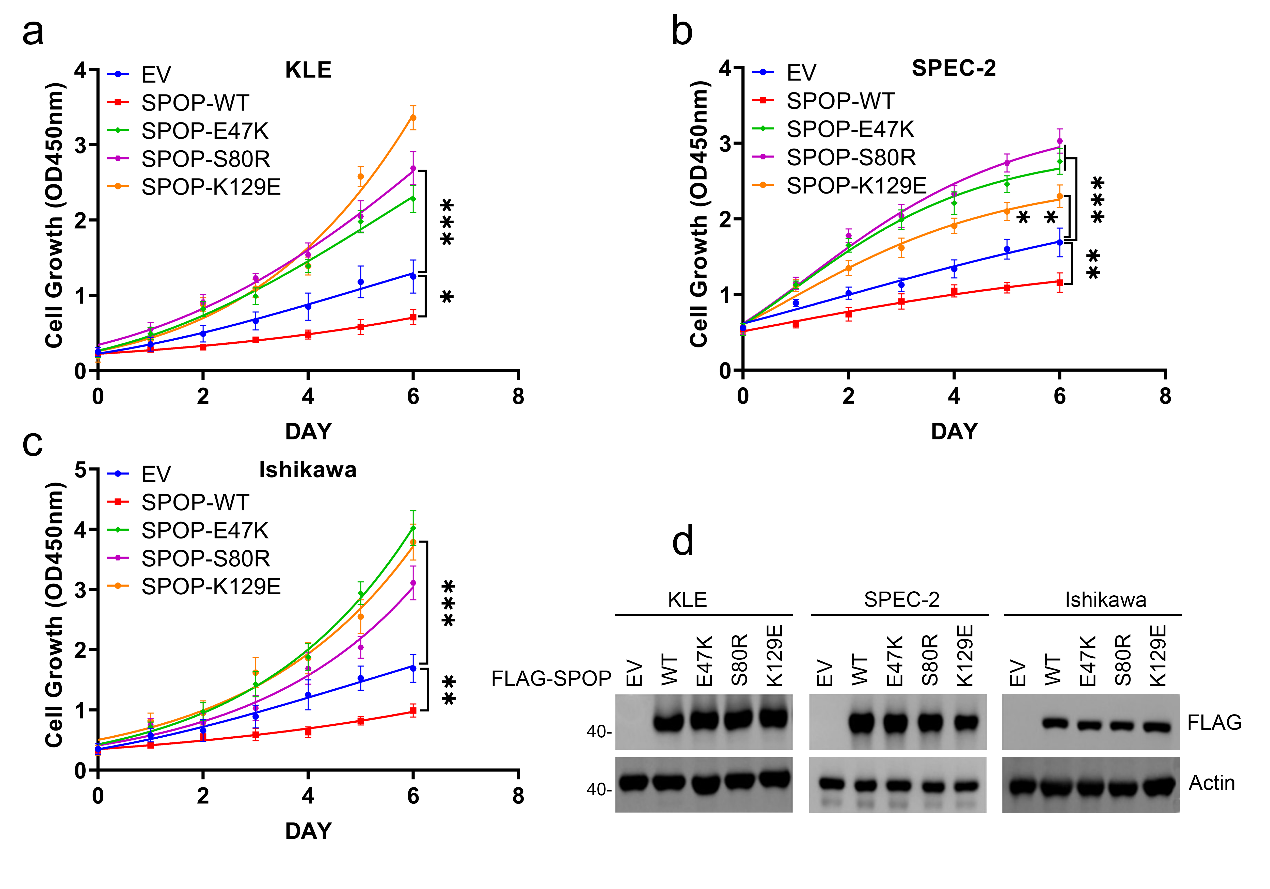
**Figure S4. The impact of SPOP-WT and EC-associated SPOP mutants on the growth of three EC cell lines.**

(a-c) CCK-8 assays in KLE (a), SPEC-2 (b), and Ishikawa (c) cells stably overexpressing EV, SPOP-WT or EC-associated SPOP mutants. Data are shown as the means ± SD (n=4).

The *p-values* were calculated using the Two-way ANOVA test in (a-c). *p<0.05, **p<0.01, ***p<0.001, ****p<0.0001.

(d) Western blot of the indicated proteins in WCL from KLE, SPEC-2, and Ishikawa cells stably overexpressing EV, SPOP-WT, or EC-associated SPOP mutants. **Table S1. Sequence information.**

| **Sequences of sgRNAs** | | |
| --- | --- | --- |
| **Gene** | **No.** | **Sequence** |
| SPOP | #1 | CAAGCTTACCCTCTTCTGCG |
|  | #2 | GTCACTTTACCTGTTACTGG |
| BRAF | #1 | GAGGCCCTATTGGACAAATT |

| **Sequences of shRNAs** | | |
| --- | --- | --- |
| **Gene** | **No.** | **Sequence** |
| SPOP | #1 | CCGGCAAGGTAGTGAAATTCTCCTACTCGAGTAGGAGAATTTCACTACCTTGTTTTTT |
|  | #2 | CCGGCACAAGGCTATCTTAGCAGCTCTCGAGAGCTGCTAAGATAGCCTTGTGTTTTTT |

| **Primers for amplification of sgRNA-targeted sequence of SPOP/BRAF gene** | | | |
| --- | --- | --- | --- |
| **Gene** | **No.** | **F:5'-3'** | **R:5'-3'** |
| SPOP | #1 | GTTGTGGCTTTGATCTGGTTTTTG | ATTTGTGCAGCACTACTCCACTTG |
|  | #2 | CCCTCTCTTGAGTCTGATTTCCA | AGAGTTGAACAAAGAGGAGAACAT |
| BRAF | #1 | TTTGAGGAACACTGGCAGTTACTG | CTCTTCCCAAATCTATTCCTAATC |

| **Primer sequence information** | | |
| --- | --- | --- |
| **Gene** | **F:5'-3'** | **R:5'-3'** |
| SPOP | ATGTCAAGGGTTCCAAGT | TTAGGATTGCTTCAGGCG |
| SPOP-ΔNLS | ATGTCAAGGGTTCCAAGT | TTATCCCAGAAAAGGGCACTG |
| BRAF | ATGGCGGCGCTGAGCGGT | TCAGTGGACAGGAAACGC |
| BRAF-ΔSBC | TCTGCATCAATGGATACC TCTTCTAGCCTTTCAGTG | CACTGAAAGGCTAGAAGA GGTATCCATTGATGCAGA |
| BRAF-V120A | ACCGCTACATCTTCTTCC | GGAAGAAGATGTAGCGGT |
| BRAF-T121A | GTTGCATCTTCTTCCTCT | AGAGGAAGAAGATGCAAC |
| BRAF-S122A | ACAGCTTCTTCCTCTTCT | AGAAGAGGAAGAAGCTGT |
| BRAF-S123A | TCTGCTTCCTCTTCTAGC | GCTAGAAGAGGAAGCAGA |
| BRAF-S124A | TCTGCCTCTTCTAGCCTT | AAGGCTAGAAGAGGCAGA |
| BRAF-T121I | TCAATGGATACCGTTATATCTTCTTCCTCTTCT | AGAAGAGGAAGAAGATATAACGGTATCCATTGA |
| BRAF-S122Y | ATGGATACCGTTACATATTCTTCCTCTTCTAGC | GCTAGAAGAGGAAGAATATGTAACGGTATCCAT |

**Table S2. Antibody and recombinant protein information.**

| **REAGENT or RESOURCE** | **Source** | **IDENTIFIER** | **Application/Dilutions** |
| --- | --- | --- | --- |
| Anti-SPOP | proteintech | 16750-1-AP | IB: 1:1000 |
| Anti-ARAF | Abcam | ab200653 | IB: 1:1000 |
| Anti-BRAF | Abclonal | A2434 | IB: 1:1000 |
| Anti-BRAF | Abcam | ab33899 | IB: 1:1000 |
| Anti-CRAF | Abclonal | A19638 | IB: 1:1000 |
| Anti-BRD4 | BETHYL | A301-985A100 | IB: 1:1000 |
| Anti-Caprin1 | proteintech | 15112-1-AP | IB: 1:1000 |
| Anti-HRAS | Abclonal | A7901 | IB: 1:1000 |
| Anti-MEK1 | Abcam | ab32091 | IB: 1:1000 |
| Anti-MEK2 | Abcam | ab32517 | IB: 1:1000 |
| Anti-ERK1/2 | CST | 4695T | IB: 1:1000 |
| Anti-MSK1 | Abclonal | A5699 | IB: 1:1000 |
| Phospho-ERK1/2 | Abcam | ab76299 | IB: 1:5000 |
| Phospho-MEK1/2 | CST | 9154T | IB: 1:1000 |
| Phospho-MSK1 | CST | 9595T | IB: 1:1000 |
| Anti-GAPDH | proteintech | 60004-1-Ig | IB: 1:10000 |
| Anti-FLAG | MBL | M185-7 | IB: 1:5000 |
| Anti-FLAG | CST | 14793S | IB: 1:5000 |
| Anti-Myc | MBL | M192-7 | IB: 1:5000 |
| Anti-HA | MBL | M180-7 | IB: 1:5000 |
| Anti-K27-linkage Specific Polyubiquitin | Abclonal | A18202 | IB: 1:1000 |
| Anti-K29-linkage Specific Polyubiquitin | Abclonal | A18198 | IB: 1:1000 |
| Anti-K48-linkage Specific Polyubiquitin | Abclonal | A3606 | IB: 1:1000 |
| Anti-K63-linkage Specific Polyubiquitin | CST | D7A11 | IB: 1:1000 |
| Creatine phosphokinase | SIGMA | Cat# 9001-15-4 |  |
| Creatine phosphate | MACKLIN | Cat# C916918 |  |
| UBE1 | Ubiquigent | Cat# 61-0001 |  |
| UBE2D1 (UbcH5a) | Ubiquigent | Cat# 62-0010 |  |
| UBE2D2 (UbcH5b) | Ubiquigent | Cat# 62-0012 |  |
| CUL3/RBX1 | Ubiquigent | Cat# 63-1003 |  |
| APP-BP1/UBA3 | Ubiquigent | Cat# 61-0006 |  |
| UBE2M (Ubc12) | Ubiquigent | Cat# 62-0048 |  |
| NEDD8 | Ubiquigent | Cat# 60-0009 |  |
| DCNL2 | Ubiquigent | Cat# 63-200 |  |

**Table S3. Cell cultures, chemicals and Kit.**

| **REAGENT or RESOURCE** | **Source** | **IDENTIFIER** |
| --- | --- | --- |
| DMEM | Gibco | Cat# 11960044 |
| Fetal Bovine Serum | Gibco | Cat# 10099 |
| Penicillin-Streptomycin | Invitrogen | Cat# 15070063 |
| EGF | novoprotein | Cat# C029 |
| Puromycin | Selleckchem | Cat# S7417 |
| Doxycycline(Dox) | Selleckchem | Cat# S5159 |
| MLN4924 | Apexbio | Cat# B1036 |
| MG132 | Selleckchem | Cat# S2619 |
| Puromycin | Sigma | Cat# P8833 |
| Trizol | Thermo Fisher | Cat# 14496026 |
| cycloheximide | MCE | Cat# HY-B0713 |
| FLAG peptide | ChinaPeptides | Cat# 04010006736 |
| HA peptide | Sigma | Cat# 26184 |
| ChamQ SYBR qPCR Master Mix | Vazyme Biotech | Cat# Q311 |
| Phanta Max Super-Fidelity DNA Polymerase | Vazyme Biotech | Cat# P505 |
| KOD-Plus-Mutagenesis Kit | TOYOBO | Cat# SMK-101 |
| Cell Counting Kit-8 | Beyotime Technology | Cat# C0037 |
| ClonExpress II One Step Cloning Kit | Vazyme Biotech | Cat# C112 |
| Anti-FLAG M2 | SIGMA | Cat# SLCD1942 |
